# Macrophage Gene Expression Associated with Remodeling of the Prepartum Rat Cervix: Microarray and Pathway Analyses

**DOI:** 10.1371/journal.pone.0119782

**Published:** 2015-03-26

**Authors:** Abigail E. Dobyns, Ravi Goyal, Lauren Grisham Carpenter, Tom C. Freeman, Lawrence D. Longo, Steven M. Yellon

**Affiliations:** 1 Center for Perinatal Biology, Loma Linda University School of Medicine, Loma Linda, CA, 92350, United States of America; 2 Departments of Basic Sciences, Loma Linda University School of Medicine, Loma Linda, CA, 92350, United States of America; 3 Division of Physiology, Pediatrics, Loma Linda University School of Medicine, Loma Linda, CA, 92350, United States of America; 4 The Roslin Institute and Royal (Dick) School of Veterinary Studies, University of Edinburgh, Easter Bush, Midlothian, Edinburgh, EH25 9RG, United Kingdom; University of Jaén, SPAIN

## Abstract

As the critical gatekeeper for birth, prepartum remodeling of the cervix is associated with increased resident macrophages (Mφ), proinflammatory processes, and extracellular matrix degradation. This study tested the hypothesis that expression of genes unique to Mφs characterizes the prepartum from unremodeled nonpregnant cervix. Perfused cervix from prepartum day 21 postbreeding (D21) or nonpregnant (NP) rats, with or without Mφs, had RNA extracted and whole genome microarray analysis performed. By subtractive analyses, expression of 194 and 120 genes related to Mφs in the cervix from D21 rats were increased and decreased, respectively. In both D21 and NP groups, 158 and 57 Mφ genes were also more or less up- or down-regulated, respectively. Mφ gene expression patterns were most strongly correlated within groups and in 5 major clustering patterns. In the cervix from D21 rats, functional categories and canonical pathways of increased expression by Mφ gene related to extracellular matrix, cell proliferation, differentiation, as well as cell signaling. Pathways were characteristic of inflammation and wound healing, e.g., CD163, CD206, and CCR2. Signatures of only inflammation pathways, e.g., CSF1R, EMR1, and MMP12 were common to both D21 and NP groups. Thus, a novel and complex balance of Mφ genes and clusters differentiated the degraded extracellular matrix and cellular genomic activities in the cervix before birth from the unremodeled state. Predicted Mφ activities, pathways, and networks raise the possibility that expression patterns of specific genes characterize and promote prepartum remodeling of the cervix for parturition at term and with preterm labor.

## Introduction

Remodeling of the cervix plays an important role as the gatekeeper for birth. Morphological transformations associated with softening of the cervix occur in advance of the transition to a contractile phenotype by the uterine myometrium [1]. In the cervix of women at term, evidence suggests local inflammatory processes are enhanced because of an increased presence of leukocytes, specifically macrophages (Mφ) and neutrophils [2,3], as well as reduced cell nuclei density, an indication of hypertrophy and edema [4,5]. In women, these processes occur without a fall in systemic progesterone concentrations. Similarly in rodents during pregnancy, prepartum inflammatory processes and structural remodeling of the cervix occur before term near the peak of serum progesterone concentrations [5–7]. Within 3–5 days before term, cervical softening is characterized by hypertrophy and edema, i.e., reduced cell density, extracellular matrix degradation, i.e., decreased collagen content and structure, and increased residency by leukocytes [8–10]. Moreover, proinflammatory signals, complement activation, transcription factor regulation, and activities by various enzyme, are temporally coincident with the transition from softening to ripening [11,12].

Little is known about molecules and network pathways that mediate the remodeling process in the prepartum cervix. Molecular studies have focused on late pregnancy and near term. In peripartum women in labor, compared to those not in labor, increased expression of genes for proinflammatory chemokine and interleukin signaling, cellular movement, extracellular matrix degradation, and immune cell-mediated inflammation are found in the cervix [13]. Similar processes were found in pools of cervix from mice during the 4 days preceding birth [14], well after the remodeling process has begun. Other studies have focused on treatment effects, but not on molecules related to structure of the cervix [15–17]. Thus, the present study focused for the first time on the transcriptome of the prepartum compared to unremodeled rat cervix to determine if a network of genes constituted a critical inflammatory pathway for remodeling the cervix.

Previous studies also indicate that differential gene expression in the peripartum cervix reflects functions by immune cells and, from other tissues, inflammatory processes linked to Mφ activities [18–21]. The census of Mφs increases several-fold before term compared to that earlier in pregnancy before remodeling in mice and rats [8,22,23]. Thus, the major objective of this study was to test the hypothesis that a novel Mφ transcriptome and gene network distinguishes the late remodeled prepartum from unremodeled nonpregnant cervix. The findings suggest that differential regulation of clusters of genes, unique to the prepartum cervix, and in common with the unremodeled cervix in nonpregnant rats, may contribute to the remodeling process in preparation for birth.

## Methods

### General procedures

The Loma Linda University Institutional Animal Care and Use Committee approved all experimental procedures (OSR# 88036), which conformed to the National Research Council standards for care and use of laboratory animals. Adult female non-pregnant (NP proestrus based upon vaginal smears; n = 6) and pregnant Sprague Dawley rats on day 21 postbreeding (D21, typically the day before birth according past experience given breeder specifications that mating is day 0; n = 6) were obtained from Harlan Laboratories (Indianapolis, IN) and housed in the vivarium with free access to food and water under 12 h of light/day (on 0700–1900h, PST). Unless noted, reagents were obtained from Sigma Chemical Co (St. Louis, Mo).

### Processing of cervix

Immediately following CO2 asphyxiation, rats were perfused though the heart with warm saline to flush out blood and systemic cells. The cervix was excised and carefully trimmed to exclude vaginal folds, adherent fat, as well as the confluence of uterine horns. The isolated cervix was rinsed (1 ml of 0.1M phosphate buffered saline), minced with scissors, and cells dispersed (1mg/ml Collagenase B with DNAase in 25 mM HEPES-HBSS at 37° C with agitation by pipette every 0.5 h for 1.5 h). The suspension, which contains a variety of cervix cell types, was passed through a 70 μm filter, centrifuged, washed and, recentrifuged (10 min at 1200 rpm at 4° C), before the cell pellet was resuspended in 2 ml HEPES-HBSS. Isolated cervix cells from each of 3 individual rats in the NP and D21 groups were flash frozen at -80C in liquid nitrogen and stored for microchip analysis of gene expression (described below). Cells from the cervix of the remaining 3 rats in each group were subjected to a magnetic bead separation procedure to remove Mφs. Briefly, a 5 μl aliquot of the final cell suspension was mixed with 5 μl trypan blue and cell counts determined with a hematocytometer. An aliquot of the Mφ-specific ED1 antibody (1μg/10^6^ cells in 0.1 ml; Serotec, Oxford, UK) was added to the cervix cell suspension. The suspension was incubated for 10 min at 8° C, centrifuged, then the cell pellet washed twice in HEPES-HBSS (2 ml/10^7^ cells). As per instruction, the cell pellet was resuspended (about 10^7^cells/ml at 8° C) and a 0.025 ml aliquot of Dynabeads in HEPES-HBSS was added (0.1ml of beads solution/10^6^ cells; DynaMag, Invitrogen Inc). After 30 min of gently rotation and agitation at 10 min intervals, each vial was placed next to a magnet apparatus for 2 min to quarantine ED-1 bound Mφs. The Mφ-depleted supernatant was decanted into a microcentrifuge tube, flash frozen, and store at -80°C for gene expression analyses. The remaining pellet with ED-1 bound Mφs did not provide sufficient RNA for consistent or accurate microarray analyses. To confirm Mφ depletion, aliquots of dispersed cervix cells from NP and D21 postbreeding rats, just prior to and after Dynabeads magnetic separation of ED-1 bound Mφs, were processed by flow cytometry and data from each run analyzed using the same gating protocol using FloJo software. This approach eliminated more than 96.75% of all Mφs from dispersed cervix. Moreover, flow cytometry results indicated that ED-1 labeled Mφs were increased in prepartum vs NP cervix, a confirmation of previous findings for F4/80-stained Mφs in mice [23].

Frozen samples from four groups of dispersed cells from the cervix of NP rats with or without Mφs, as well as from D21 prepartum rats or prepartum cervix without Mφs were sent to GenUs Biosystems (Chicago, IL). Briefly, samples were lysed in Tri-reagent and total RNA isolated using phenol/chloroform extraction followed by purification over spin columns (reagents from Ambion, Austin, TX). The concentration and purity of total RNA was measured by spectrophotometry at OD260/280 and the quality of the total RNA sample was assessed using an Agilent Bioanalyzer with the RNA6000 Nano Lab Chip (Agilent Technologies, Santa Clara, CA). Labeled cRNA was prepared by linear amplification of the Poly(A)+RNA population within the total RNA sample. Briefly, 1 μg of total RNA was reverse transcribed after priming with a DNA oligonucleotide containing the T7 RNA polymerase promoter 5’ to a d(T)24 sequence. After second-strand cDNA synthesis and purification of double-stranded cDNA, *in vitro* transcription was performed using T7 RNA polymerase. The quantity and quality of the labeled cRNA was assayed by spectrophotometry and the Agilent Bioanalyzer. For the standard genome analysis, 1 μg of the purified cRNA from each individual cervix was fragmented to uniform size and applied to the Rat Gene Expression 4x44K v3 microarray chips in hybridization buffer (Agilent Technologies). Arrays were hybridized at 65° C for 17h in a shaking incubator and washed at 37° C for 1 min. Rinsed and dried arrays were scanned at 5 μm resolution with an Agilent G2565 Microarray Scanner (Agilent Technologies). Data from scanned images of arrays were processed and analyzed with Feature Extraction and GeneSpring GX v7.3.1 software (Agilent Technologies).

### Correlations, functional categories, canonical pathways, and Network analyses

Raw optical intensity data were normalized to the 75^th^ percentile of each array. Fold-changes and p values for gene expression were determined by comparing the average intensity from the entire cervix in rats on the day before expected birth (late remodeled) versus unremodeled state, i.e., D21 vs NP. To identify Mφ genes, expression of genes from dispersed cervix, with or without Mφs, were compared in D21 to NP rats. The threshold for significant differential gene expression was p<0.01 with 2-fold increase or decrease. Mφ genes were sorted based on 1) exclusive to D21, 2) exclusive to NP, or 3) non-exclusive in common to D21 and NP groups.

To evaluate genomic expression, expression patterns of Mφ genes in the cervix of individuals within groups were evaluated using Biolayout *Express* ^3D^ (http://www.biolayout.org/) [24,25]. For this analysis, genes were uploaded with the minimum threshold of significance (p<0.01 and fold >2 or <-2). These data for individuals were transposed and run to identify group clusters. To identify major clusters of genes that were related to Mφs, irrespective of group, the entire dataset from all 4 groups was uploaded and thresholds set for Pearson’s correlation (R> = 0.9) and a minimum intensity at 1 to generate a network graph. The Markov clustering algorithm (MCL settings: inflation = 1.7, minimum cluster = 3) was used to identify clusters of up regulated Mφ genes. This list of 27 gene clusters was extracted and reanalyzed as before, but only with data from whole cervix (with Mφ) to generate an exclusive Mφ gene correlation network. Limited numbers of down regulated Mφ genes precluded further Biolayout *Express* ^3D^ analyses.

Data were further analyzed by Ingenuity Pathway Analysis (IPA, Ingenuity Systems, Redwood City, CA) to identify functional categories and canonical pathways of Mφ genes in the cervix from the D21 group. Mφ genes that were exclusively regulated in the D21 group and most divergently regulated in common in D21 prepartum and NP group (ratio of >1.5-fold or <0.75-fold, D21 vs NP groups) were further analyzed to identify relevant functional pathways with a significant proportion of genes predicted to be activated or inhibited with respect to the referenced IPA literature database (p<0.01 and Z-Score>2 or <-2). Based upon the number of significantly regulated Mφ genes, a ranked table of canonical pathways was identified (p<0.05). Finally, a network of possible interactions among prepartum Mφ and other relevant genes was constructed.

### Quantitative Real-Time PCR validation

To validate the results of the microarray analysis, we chose highly regulated genes (FCN1, CD163, CCR2, MPEG1, COL4A5, HAS2) for analysis using real time PCR. Using the same probe sequences as those on the microarray chip, we designed primers with the use of Primer 3 web-based software (http://frodo.wi.mit.edu/primer3/). The primers were synthesized by Integrated DNA technologies (Coralville, CA). Total RNA (1 μg per reaction) was reverse transcribed using Quantitect reverse transcriptase kit (Qiagen, Valencia, CA). Relative expression was normalized to 18S RNA and fold-changes were calculated using the ΔΔCt method with normalization of individual PCR efficiencies. Samples were analyzed on the Roche LightCycler 1.5 (Roche, Indianapolis, IN).

### Statistics

To compare individual expression values across arrays, the raw intensity data from each gene was normalized to the 75^th^ percentile intensity of each array. Only genes with values greater than background intensity for all samples within individuals were further analyzed. Differences in mean intensity of expressed genes between the cervices from rats in D21 or NP groups, with or without macrophages, respectively, met or exceeded criteria of 2-fold change with p<0.01 (Welch’s t-test). Statistical significance in the real-time PCR data was assessed by one-way analysis of variance and the *post-hoc* Newman-Keuls test.

## Results

Analyses of microarray data in whole cervix of prepartum day 21 rats indicated that expression of 255 genes increased and 128 genes decreased relative to that in the NP group (Tables 1 and 2, respectively; p<0.01, contain fold differences ≥6 or ≤-5). A complete list of regulated genes has been uploaded (http://www.ncbi.nlm.nih.gov/geo/). Of the genes with most increased or decreased expression in the cervix from D21 versus NP rats, 34 were enhanced >6-fold and 22 were suppressed >5-fold. Some of the most highly regulated genes included CFB, CD163, PIGR, CHRND, PTGDR, HIF1α, and HOXA9—transcriptional messages that are indicative of effector molecules from immune cells and the focus of further analyses.

**Table 1:** Increased expression of genes in whole cervix from prepartum D21 postbreeding versus nonpregnant rats (p<0.01; n = 3/group).

Symbol	Entrez Gene Name	Fold Change
O3FAR1	Omega-3 fatty acid receptor 1	48
SCGB3A1	Secretoglobin, family 3A, member 1	25
Gkn3	Gastrokine 3	24
CHRND	Cholinergic receptor, nicotinic, delta (muscle)	20
SOX9	SRY (sex determining region Y)-box 9	12
CD163	CD163 molecule	12
GALNT6	UDP-N-acetyl-alpha-D-galactosamine:polypeptide N-acetylgalactosaminyltransferase 6	12
CAPN5	Calpain 5	11
UCP3	Uncoupling protein 3 (mitochondrial, proton carrier)	11
CFB	Complement factor B	9
ANO7	Anoctamin 7	9
CLCA4	Chloride channel accessory 4	8
BCAS1	Breast carcinoma amplified sequence 1	8
CREG1	Cellular repressor of E1A-stimulated genes 1	8
ALPL	Alkaline phosphatase, liver/bone/kidney	8
ENTPD8	Ectonucleoside triphosphate diphosphohydrolase 8	7
PTGDR	Prostaglandin D2 receptor (DP)	7
UNC5B	Unc-5 homolog B (C. elegans)	7
KRT20	Keratin 20	7
EPS8L3	EPS8-like 3	7
SCN1B	Sodium channel, voltage-gated, type I, beta subunit	7
LGALS12	Lectin, galactoside-binding, soluble, 12	7
GJC2	Gap junction protein, gamma 2, 47kDa	7
EPHA2	EPH receptor A2	7
ATP8B3	ATPase, aminophospholipid transporter,class I, type 8B, member 3	6
Ces2b	Carboxylesterase 2C	6
PIGR	Polymeric immunoglobulin receptor	6
TBC1D2	TBC1 domain family, member 2	6
CDK18	Cyclin-dependent kinase 18	6
PLA2G2A	Phospholipase A2, group IIA (platelets, synovial fluid)	6
FOS	FBJ murine osteosarcoma viral oncogene homolog	6
DSG2	Desmoglein 2	6
UPK1B	Uroplakin 1B	6
CLDN7	Claudin 7	6

**Table 2:** Decreased expression of genes in whole cervix from prepartum D21 postbreeding versus nonpregnant rats (p<0.01; n = 3/group).

Symbol	Entrez Gene Name	Fold Change
UNC45B	Unc-45 homolog B (C. elegans)	−17
TNNC2	Troponin C type 2 (fast)	−16
ZP2	Zona pellucida glycoprotein 2 (sperm receptor)	−13
KRT4	Keratin 4	−10
BEX4	Brain expressed, X-linked 4	−10
PCP4L1	Purkinje cell protein 4 like 1	−10
THEM5	Thioesterase superfamily member 5	−8
HOXD10	Homeobox D10	−8
Gsta4	Glutathione S-transferase, alpha 4	−8
GDF5	Growth differentiation factor 5	−7
TNNI2	Troponin I type 2 (skeletal, fast)	−7
CASQ1	Calsequestrin 1 (fast-twitch, skeletal muscle)	−7
HOXD9	Homeobox D9	−7
PMEL	Premelanosome protein	−6
TRPC3	Transient receptor potential cation channel, subfamily C, member 3	−6
MOXD1	Monooxygenase, DBH-like 1	−6
MMP16	Matrix metallopeptidase 16 (membrane-inserted)	−6
CHRDL1	Chordin-like 1	−6
AMN	Amnionless homolog (mouse)	−5
MYOM1	Myomesin 1, 185kDa	−5
GZMM	Granzyme M (lymphocyte met-ase 1)	−5
HOXA9	Homeobox A9	−5

**Fig 1:**
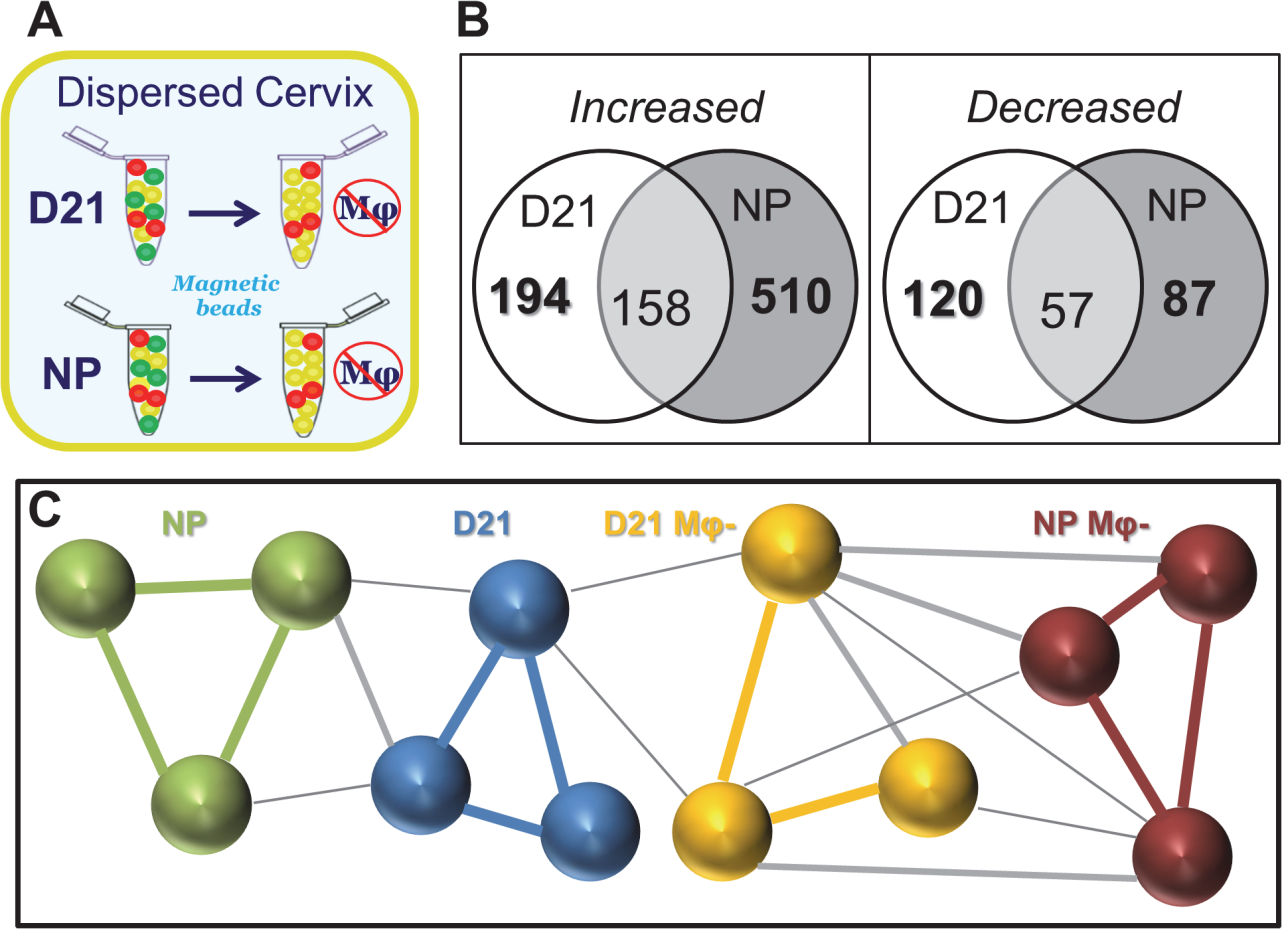
Subtractive approach in which macrophages were depleted from dispersed cervix from prepartum and nonpregnant mice to identify differential gene expression by macrophages. **A**. Cervix from perfused adult female Sprague-Dawley rats, on prepartum day 21 post-breeding (D21) or non-pregnant (NP) was carefully trimmed and dispersed (n = 6 each). Mφs were removed by magnetic bead separation from 3 rats in each group as described in detail in Methods. **B**. Venn diagram of differentially expressed Mφ genes in the cervix from prepartum (D21) or nonpregnant (NP) rats. Genes were exclusively increased or decreased in cervices from D21 or NP rats, except in the overlap region which indicates genes that were increased or decreased in cervices from both D21 and NP rats. No gene whose expression increased or decreased in the cervix from D21 or NP groups then decreased or increased in the cervix from NP or D21 groups, respectively. Number is the average of differentially expressed Mφ genes in whole divided by Mφ -depleted cervix/group (p<0.01, fold >2 or <-2; n = 3 rats). **C**. Pearson correlation of clustering patterns of gene expression in cervix of individual rats in NP and D21 groups with or without macrophages (Mφ-). Lines connecting microarray analysis for each individual indicates R>0.9 (group specific colors), R = 0.851–0.89 (grey), or R = 0.8–0.85 (thin grey).

To identify gene expression specifically related to Mφs, microarray data were compared from the whole cervix of D21 and NP rats to that following depletion of Mφ (Fig. 1 A and B). Expression of 194 and 120 genes were found to increase and decrease, respectively, in the cervix from prepartum D21 rats. Thus relative to whole cervix changes, macrophages accounted for 61 up- and 4 down-regulated genes. Based upon fold-difference and p-value, the 32 most up-regulated Mφ genes that were exclusively in the prepartum cervix (Table 3) included proinflammatory markers of activation MHCII CD300, TLR7, and CCR2, extracellular matrix degradation (COL4A5), as well as wound healing markers CD206 (MRC1), CD163, F13A1 according to DAVID analysis (http://david.abcc.ncifcrf.gov ). The 31 most down-regulated Mφ genes that were exclusively in the cervix from D21 rats (Table 4) included molecules linked to vasoconstriction (EDN2), cell growth (FOSL1, EGF4, FOSB), and intercellular adhesion (COL17A1). In addition, expression of Mφ genes in both prepartum and NP rats featured up-regulation of proinflammatory markers (CSFR1, CXCL13, CCR1, several HLA components, CREM, MMP12, and S5AR1), as well as down-regulation of message in signaling pathways (WNT10A, JUN/JUNB, BCL11B). Other key differentially regulated molecules were ADAM10, CCL5, F3, and PVRL2.

**Table 3:** Increased expression of Mφ genes exclusively in prepartum D21 rat cervix (p<0.01; n = 3/group).

Symbol	Entrez Gene Name	Function	Fold
FCN1	Ficolin 1 (collagen/fibrinogen domain)	Collagen-like domain, lectin activity; Clusters 2 & 8; ECM	77
GPR34	G protein-coupled receptor 34	Cell Signaling; SIG	68
CD206	Mannose receptor, C type 1	Cluster 2; ECM	67
CD300C	CD300c molecule	On specific immune cells & cell lines	57
MS4A4A	Membrane-spanning 4-domains, subfam A	Cluster 2	44
CD163	CD163 molecule	Scavenger receptor activity; Cluster 2	39
F13A1	Coagulation factor XIII, A1 polypeptide	Involved in wound healing; Cluster 2; ECM	32
PTPRC	Protein tyrosine phosphatase, receptor type C	Humoral&cell-mediated immunity; Cluster 1; ECM	30
RGS18	Regulator of G-protein signaling 18	Signal Transduction Inhibition; SIG	28
CPA3	Carboxypeptidase A3 (mast cell)	Catalyst of C-terminal amino acid release	27
Mcpt1	Mast cell protease 1	Chymotripsin-like activity, INFL	27
EVI2A	Ecotropic viral integration site 2A	Membrane complex protein receptor, Cluster 1	24
HLA-DRB1	MHCII, DR beta 1	Chronic inflammation; INFL	22
CCR2	Chemokine (C-C motif) receptor 2	MCP-1, -3, -4 chemokines; Cluster 1; INFL	22
FAIM3	Fas apoptotic inhibitory molecule 3	Apoptosis protection and immune system processes; Cluster 2; INFL	18
NMUR2	Neuromedin U receptor 2	Selective Neuropeptide Receptor	16
SMOC1	SPARC related modular calcium binding 1	Eye and Limb development Regulation	15
SLC11A1	Solute carrier family 11 member 1	Iron and Manganese Transporter	15
ARL11	ADP-ribosylation factor-like 11	Tumor suppression, apoptosis	14
TLR7	Toll-like receptor 7	Innate immune response via cytokine secretion and inflammatory responses; INFL	13
PPYR1	Pancreatic polypeptide receptor 1	Selective Neuropeptide Receptor	13
BCMO1	Beta-carotene 15,15'-monooxygenase 1	Enzyme Responsible for Retinal Formation	12
COCH	Coagulation factor C homolog, cochlin (L. polyphemus)	Cell Shape and Motility	12
PRKCB	Protein kinase C, beta	Phorbol esters receptor (tumor promoters); Cluster 13; SIG	12
MPEG1	Macrophage expressed 1	Expressed in macrophages.	12
MYO1F	Myosin IF	Intracellular movement; Cluster 1; ECM	12
MYO1G	Myosin IG	Intracellular Movements; ECM	11
TPSAB	Tryptase alpha/beta 1	ECM	10
SYNPO2	Synaptopodin 2	Actin Binding and Bundling; ECM	10
AOAH	Acyloxyacyl hydrolase (neutrophil)	Removes secondary (acyloxyacyl-linked) fatty acyl chains from the lipid A region of bacterial LPS	10
BTK	Bruton agammaglobulinemia tyrosine kinase	B cell maturation	10
COL4A5	Collagen, type IV, alpha 5	Type IV collagen formation in glomeruli structure; Cluster 6; SIG	10

Function designations in rat derived from DAVID (http://david.abcc.ncifcrf.gov), Biolayout *Express* ^3D^ cluster analyses, and IPA (ECM = extracellular matrix, INFL = inflammation, SIG = Signaling).

**Table 4:** Decreased expression of Mφ genes exclusively in prepartum D21 rat cervix (p<0.01; n = 3/group).

Symbol	Entrez Gene Name	Function	Fold
FOSL1	FOS-like antigen 1	SIG	−52
EGR4	Early growth response 4	Transcriptional regulation; SIG	−38
SMG6	Smg-6 homolog, nonsense mediated mRNA decay factor	Replication Regulation	−28
EGR1	Early growth response 1	Transcriptional regulation; SIG	−21
UCN2	Urocortin 2	Digestion Regulation	−17
CCR9	Chemokine (C-C motif) receptor 9	Chemokine receptor; INFL	−15
FOSB	FBJ murine osteosarcoma viral oncogene homolog B	DNA binding by Jun proteins; SIG	−15
CSRNP1	Cysteine-serine-rich nuclear protein 1	Tumor Suppression	−13
FOXN1	Forkhead box N1	Keratin Transcriptional Regulation and Immune Regulation	−13
DUSP5	Dual specificity phosphatase 5	Phosphoprotein	−12
C10orf10	Chromosome 10 open reading frame 10	Transcription Factor	−11
OVOL1	Ovo-like 1(Drosophila)	Zinc Finger Transcription Factor	−10
PLEKHA6	Pleckstrin homology domain containing, family A member 6	Phospholipid Binding Protein	−10
SAP25	Sin3A-associated protein, 25kDa	Transcriptional repression; SIG	−10
Hist2h4	Histone cluster 2, H4	Core nucleosome component; SIG	−9
ACRBP	Acrosin binding protein	Acrosin zymogen packaging and condensation; ECM	−8
COL17A1	Collagen, type XVII, alpha 1	Transmembrane Protein of Type XVII Collagen; ECM	−8
TP63	Tumor protein p63	SIG	−8
TRIM29	Tripartite motif containing 29	SIG	−7
CLDN4	Claudin 4	Tight Junction-Specific Obliteration	−7
C10orf2	Chromosome 10 open reading frame 2	Mitochondrial DNA Metabolism	−6
GPRC5A	G protein-coupled receptor, family C, group 5, member A	G-Protein Signaling; SIG	−6
THEM5	Thioesterase superfamily member 5	Mitochondrial Metabolism	−6
Calcb	Calcitonin-related polypeptide, beta	Induces Vasodilation	−6
MAP3K8	Mitogen-activated protein kinase 8	Cell cycle; SIG	−5
HELZ	Helicase with zinc finger	RNA metabolism; INFL	−5
IFRD1	INF-related developmental regulator 1	Proliferation/differentiation in NGF-induced pathways; INFL	−5
FAT2	FAT tumor suppressor homolog 2 (Drosophila)	Cell Adhesion and Proliferation	−5
NPFF	Neuropeptide FF-amide peptide precursor	Morphine Modulating Peptide	−5
HAP1	Huntingtin-associated protein 1	Protein Associated with Huntington’s Disease	−5
PKMYT1	Protein kinase, membrane associated tyrosine/threonine 1	Protein Kinase Superfamily; SIG	−5

Function designations in rat derived from analyses using DAVID (http://david.abcc.ncifcrf.gov ) and IPA (ECM = extracellular matrix, INFL = inflammation, SIG = Signaling).

We used Biolayout *Express* ^3D^ to evaluate the correlation of gene expression patterns among microarray datasets. Individuals within each group had the highest Pearson correlations(R>0.9; Fig. 1C colored lines). Gene expression patterns were correlated to a lesser extent among rats in both NP and D21 groups for whole cervix or after Mφ depletion. Thus, Biolayout *Express* ^3D^ identified individuals within each of the 4 groups in the study as most correlated in gene expression patterns.

**Fig 2:**
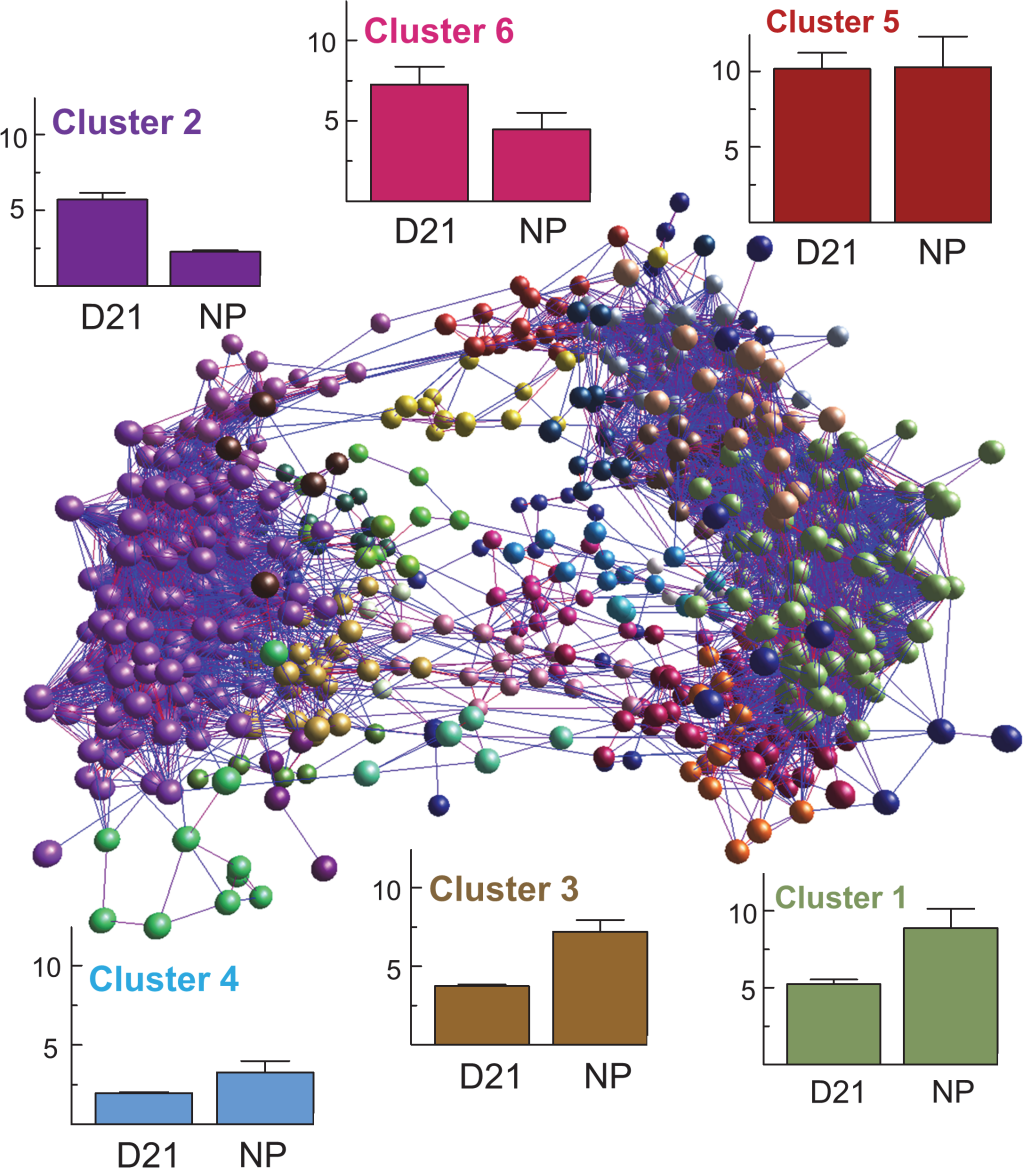
Biolayout *Express* ^3D^ analyses of Mφ gene expression and clustering analyses. Data were transposed in an analysis to identify correlations among groups based on individual gene expression levels (Pearson’s correlation of R≥0.9 and intensity >1 of dataset with fold >2 or <-2 and p<0.01). Markov clustering (MCL, inflation = 1.7) was used to identify clusters related to Mφ genes (microarray results from whole divided by Mφ -depleted cervix). These clusters were analyzed again to create the network schema with 1418 nodes and 69761 edges. Color of nodes represents membership in clusters. The 5 largest MCL clusters had distinct patterns that represent 80% of differentially regulated Mφ genes in cervix (histogram insets, average intensity ± SE, n = 3 rats/group).

Furthermore, Biolayout *Express* ^3D^ generated a network of 1418 Mφ genes with 69,761 edges of varying correlation. Based upon Markov clustering analysis a network of 6 distinct clusters of Mφ gene expression patterns accounted for 80% of all regulated message (Fig. 2; Clusters 1–6 insets). Of these, expression was up-regulated in the cervix from D21 versus NP rats in 541 Mφ genes in Cluster 1, as well as 240, 143, 121, 43, and 32 Mφ genes in Clusters 2, 3, 4, 5 and 6. Distinct expression patterns indicated Mφ genes in Clusters 2 and 6 were more enhanced in the cervix of D21 versus NP rats. By contrast, Clusters 1, 3, and 4 had greater expression in the cervix from rats in NP versus D21 groups. Cluster 5 contained Mφ genes that were equally enhanced in the cervix from D21 and NP groups. These Biolayout *Express* ^3D^ -identified unique clustering patterns of highly regulated Mφ gene expression were not predictive of a consistent functional activity when genes were cross-referenced with the DAVID database.

**Fig 3:**
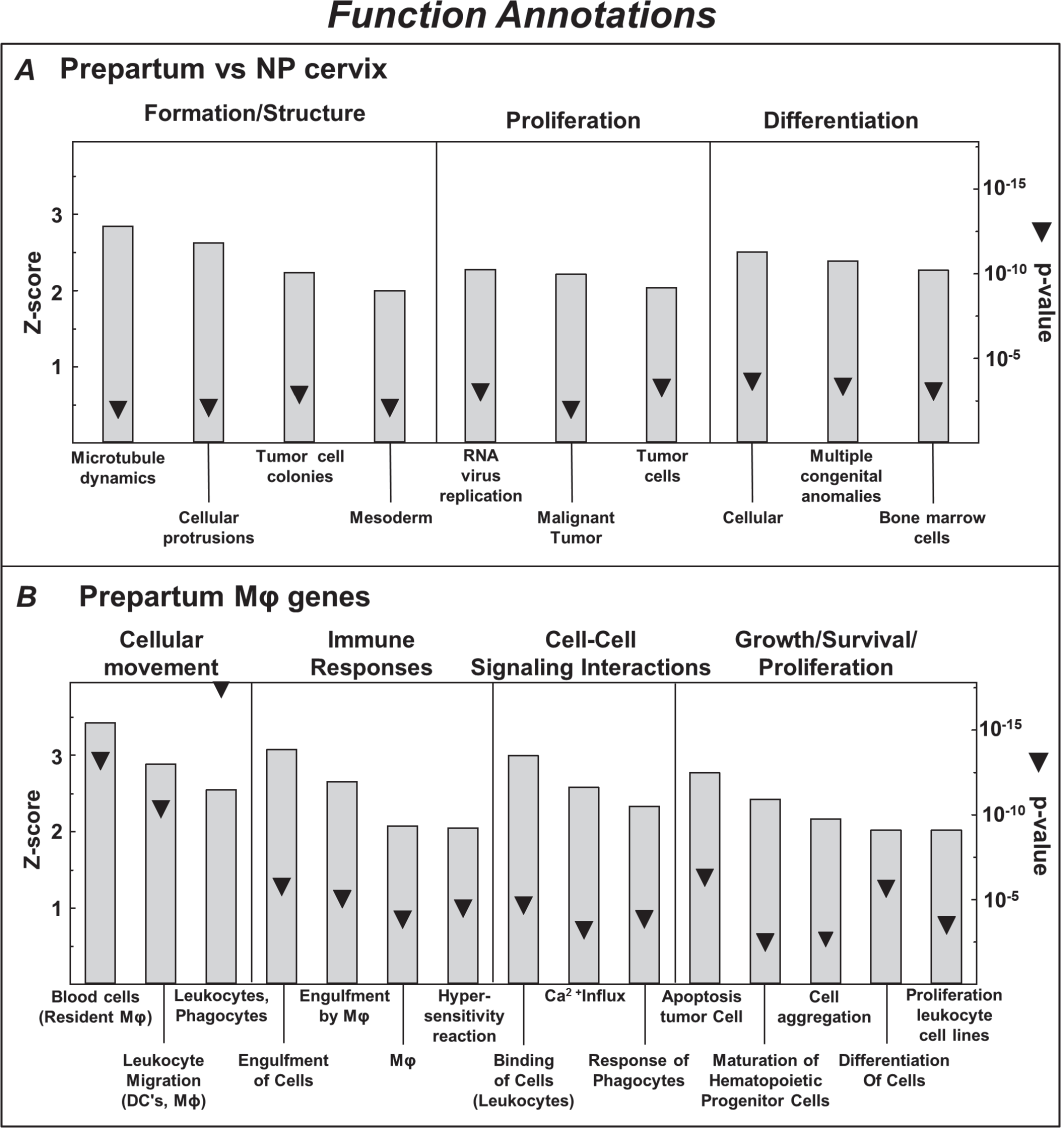
Functional annotations from Ingenuity Pathway Analysis (IPA) of increased expression of genes in the whole cervix from prepartum (D21) versus nonpregnant (NP) rats (A) and Mφ genes exclusively up-regulated in the cervix (whole vs Mφ-depleted) from prepartum rats (B). Categories of Function (Headings) and Annotations (histogram labels) consist of IPA designations based upon Z-score rank (threshold>2 estimates proportion of genes that were increased within each annotation; p < 0.01). Categories were broadened (Subheadings combined) to eliminate redundancy in IPA assignment of genes. p value is indicated by triangles. Mφ data analysis included expression of genes that were most divergently regulated, exclusively and in common, in cervices from D21 prepartum and NP groups (ratio of fold > 2 or < 2, D21 vs NP groups).

For that reason, pathway analysis was used to identify functions associated with categories of regulated genes in the prepartum D21 and NP cervix (IPA Biological Functions Core analysis). Categories of functions were broadened to be more comprehensive and eliminate redundant IPA-designated annotations with overlapping genes (Fig. 3). In the cervix from prepartum versus NP rats, Z-score and p-value were used to identify categories of cellular functions predicted to be activated, i.e., tissue formation/structure, as well as cellular proliferation and differentiation of cells (Fig. 3A). Within these categories (D21 vs NP whole cervix), the IPA database identified activated function annotations for cancer and bone marrow-derived cells. Expression of Mφ genes exclusively in the prepartum cervix (D21 whole vs Mφ -depleted cervix, Fig. 3B) reflected particular function categories related to cellular movement, proinflammatory immune responses (phagocytosis), intercellular signaling, as well as growth, survival, and proliferation.

Only a few function categories were predicted to be inhibited based on the gene expression in cervix. IPA annotated inhibited function groupings in cervix from D21 versus NP rats were organismal death, cellular apoptosis and adhesion, HDL cholesterol, and reactive oxygen species generation. For Mφ genes in the cervix from prepartum rats, predicted inhibited function categories were innate immunity, axonal guidance/seizures, and proliferation of vascular smooth muscle.

**Fig 4:**
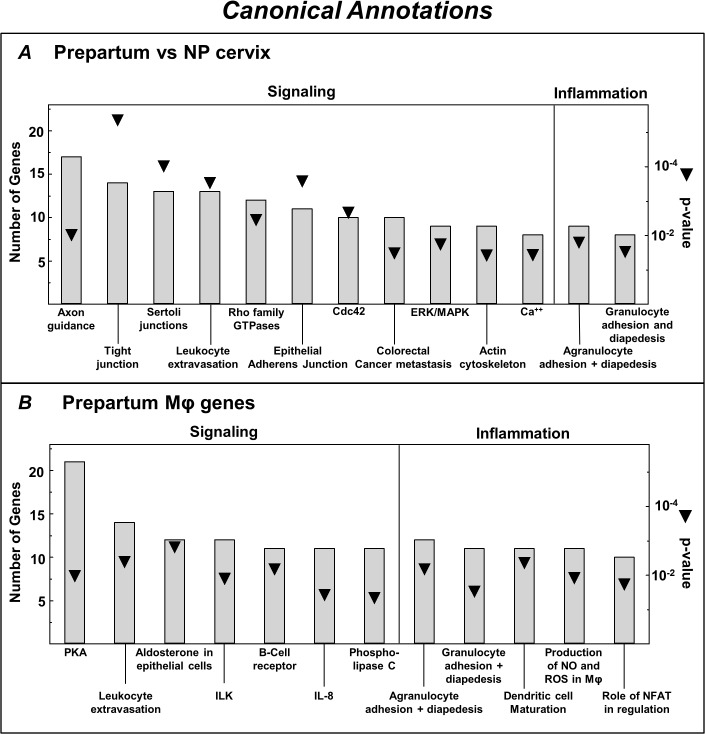
Canonical annotations from Ingenuity Pathway Analysis (IPA) of increased expression of genes in the whole cervix from prepartum (D21) versus NP rats (A) and Mφ genes exclusively up-regulated in the cervix (whole vs Mφ—depleted) from prepartum rats (B). Canonical pathways are IPA designations of rankings based on the number of genes with increased or decreased expression; p < 0.01. p value is indicated by triangles. Subheadings reflect IPA assignment of genes in common for Category annotations. Mφ data analysis included expression of genes that were most divergently regulated, exclusively and in common, in cervices from D21 prepartum and NP groups (ratio of fold >2 or < 2, D21 vs NP groups).

Analyses of differential expression of genes in cervix from prepartum and NP rats identified in certain Canonical pathways were principally related to signaling and inflammation. Specific pathways were axonal guidance, intercellular junction communication, intracellular signal transduction, granulocyte adhesion and diapedesis, and cancer-related activities (Fig. 4A). Canonical pathways that reflected regulation of Mφ genes in the prepartum cervix included immune cell extravasation, diapedesis, and adhesion, nitric oxide, and reactive oxygen species production (Fig. 4B).

In contrast to Mφ genes that were exclusively regulated in the prepartum cervix, some Mφ genes were also differentially expressed in the cervix from both prepartum and NP rats. Common to both groups, 158 and 57 genes (Fig. 1, Venn diagram intersections), were consistently, more or less, up- or down-regulated (S1 Table, Mφ regulated genes in common). Some of the most disparate genes that were higher in D21 than NP were MMP12, Fcna, CCR1, and CD4. Others that were higher in cervix from NP than D21 rats included: CLMP, SERPINF1, CD34, MRC2, and PRLP. Based upon IPA analysis (S1 Fig), categories of specific functions by Mφ in the cervix include cell movement (leukocytes), inflammation (engulfment, response of myeloid cells, activation of phagocytes), growth and survival (differentiation and quantity of leukocytes, proliferation of cells, fatty acid metabolism), and cell signaling (adhesion of blood cells, binding of cells). No inhibited Mφ functional categories were identified. Canonical pathways related to the presence of Mφs in the prepartum cervix were cellular signaling by Cdc42, ILK, and protein kinase θ, as well as those related to aspects of inflammation, dendritic cell maturation, and agranulocyte adhesion and diapedesis (S1 Fig, panel B). Additional analyses were not done on Mφ genes that were exclusively up- or down-regulated in NP rats (S2 Table and S3 Table, respectively).

## Discussion

**Fig 5:**
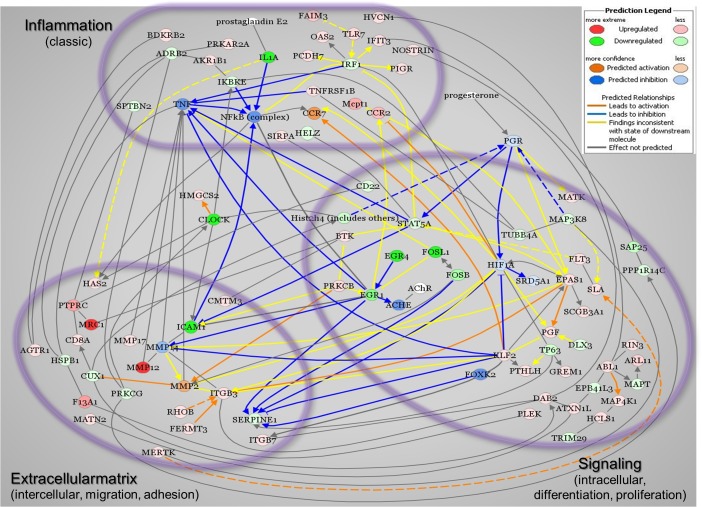
Proposed network of pathways that reflect expression of Mφ genes in the prepartum cervix based upon Ingenuity Pathway Analysis (IPA). Network includes Mφ genes exclusively regulated in the cervix from D21 rats and those regulated in both in D21 prepartum and NP groups, as well as known key molecules. Red signifies up-regulated genes and green indicates down-regulated expression. IPA drawn lines predicted activation (orange) or inhibition (blue) of gene expression between key molecules. Other lines indicate no known relationship (black) or uncertainty about relationship (yellow). Color intensity indicates relative expression based upon p-value or prediction intensity. Groups of genes are clustered into Inflammation, Extracellular matrix, or Signaling based on annotations provided by IPA.

The objective of the present study was to identify differentially expressed genes and genomic pathways specifically related to Mφs in the cervix late in the remodeling process. In support of the hypothesis, major findings of this study were that a limited number of genes related to Mφs are up- or down regulated exclusively in the prepartum D21 on the day before birth compared to that in the unremodeled cervix before pregnancy. In addition, some Mφ genes in the cervix from both prepartum D21and NP rats were consistently either up- or down-regulated. Such genomic expression differences reflect broad changes associated with pregnancy, a subset of which may be specifically related to cervical remodeling. Overall, the majority of differentially regulated Mφ genes had predicted activities and canonical pathways that reflect morphological or structural transformations, including cellular proliferation, extracellular matrix reorganization, and cellular communication. Though conclusions based upon 3 rats/group may be a limitation of the study to resolve the spectrum of possible Mφ activities that distinguish a prepartum from unremodeled cervix, the evidence suggests that a novel network of molecules, associated with the extracellular matrix, as well as proinflammatory and signal transduction processes, may contribute to the remodeling process (Fig. 5).

The novel use of Biolayout *Express* ^3D^ and Ingenuity Pathway Analysis network analyses provides insights into the remodeling process that extends beyond current understandings. A complex clustering of Mφ genes with patterns of coexpression and functional groupings appear to characterize the prepartum versus the unremodeled cervix. Rather than indicating a specific change in expression of individual genes, evidence suggests highly connected cliques within a network of genes are collectively up- or down-regulated in the cervix by the day before birth. These Mφ genes are predictive of inflammation, extracellular matrix degradation, and signal transduction activities. Analyses of message expression patterns and networks expands our understanding of the coexpression, clustering, and connectivity of Mφ genes in the cervix near term, and raises the possibility that a unique phenotype of Mφ activities may tip the balance to accelerate fibrolytic activities and reduce fibrogenesis, e.g. Clusters 1, 3, 4 Mφ genes. Defining a molecular switch that characterizes the transition to ripening might have predictive value for when the remodeling process is advanced or delayed.

The contention that proinflammatory transcriptional molecules and pathways may be a component of the remodeling processes is consistent with a study of cervical biopsies from women in labor. Similar genes were implicated, to a limited extent, in previous studies of peripartum human and mouse cervix [13,14]. Some of these genes code for Mφ-related molecules that mediate the production of nitric oxide, reactive oxygen species, and ILK/IL-8 signaling, e.g., CD300, TLR7, and CCR2. In a broader context, up-regulation of genes that are exclusively in the peripartum cervix at term, such as CFB, PIGR, and PTGER4, are part of functional categories that include cellular movement and immune responses, as well as canonical pathways that involve leukocyte extravasation and signaling. In the present study, the identification of similar genes and pathways with the unique approach on the Mφ transcriptome related to chemokines, coagulation factors, and regulation of the extracellular matrix supports the novel hypothesis that Mφs are critical participants in remodeling processes that prepares the prepartum cervix for birth at term.

In addition to differentially regulated Mφ genes associated with proinflammatory activities, others reflect alternative wound-healing activities. Examples of this alternative phenotype include CD206, CD163, and F13A1, and COL4A5, which are associated with phagocyte activities such as engulfment and leukocyte migration. Limited numbers of predicted inhibited functional categories and pathways were related to organismal and cellular death (HOXD10 and HOXA9). The coexistence of up-regulated classic inflammatory and alternative wound-healing activities by Mφs are characteristic of fibrolytic processes in other tissues and pathologies in humans [26,27,28]. Notably absent from the dataset are molecules related to anti-inflammatory processes, IL10 and FoxP3 in particular. Such differential gene expression may be indicative of a diversity of Mφ activities within a given tissue. This concept extends beyond the M1/M2 designations for models of Mφ activities, and is consistent with the emerging perspective that a spectrum of Mφ phenotypes may coexist within the same tissue to direct local morphological, physiological, or pathophysiological transformations [19].

These analyses and findings also present the novel idea that the remodeling process may depend upon differential expression of genes in the cervix of both prepartum and nonpregnant rats. Regulation of a network of genes in common suggests an ongoing process of inflammation at term and before pregnancy (S2 Fig). Although IPA analyses of microarray data has proven useful, an important limitation is the dependence on available literature, which predominantly does not include the cervix. Even so, the overall findings are strengthened by the experimental design where systemically perfused and carefully dissected cervices exclude the larger volume of blood and immune cells present in this tissue near term compared to that before pregnancy [23]. This focus on a resident cell population and the use of Biolayout *Express* ^3D^ revealed that the highest correlation of Mφ gene expression patterns in the prepartum cervix were among rats within each group and among 6 distinct clusters. The importance of this novel finding is that a balance of diverse Mφ activities (inflammation and would healing) may define the switch to ripening by the day before birth in advance of the onset of labor.

### Summary and perspective

Analyses of Mφ genes in the prepartum cervix indicate differential expression that is predictive of a unique combination of inflammation and wound-healing activities. Implications are that these molecules and pathways contribute to the remodeling process, during which the cervix *virtually disappears* in advance of birth and then is restored postpartum to an unremodeled unscarred state. Approaches to regulate such highly divergent expression of Mφ genes might prove useful to impede certain Mφ activities (inflammation, phagocytosis, migration, and repair), and importantly, to forestall the ripening process that is advanced with preterm birth. This same goal could be achieved by promoting activities by highly expressed Mφ genes in the cervix from NP versus prepartum rats to restore extracellular matrix structure and suppress inflammation, proliferation, and growth. The present study emphasizes the value of network and cluster analyses, though functions of many highly differentially regulated genes have yet to be realized or related to categories of activities and pathways that regulate remodeling of the cervix in preparation for parturition.

## Supporting Information

S1 Fig Functional annotations and Canonical pathways for macrophage gene expressed in cervix of prepartum and nonpregnant rats. A. Functional annotation categories B. Canonical Pathways from Ingenuity Pathway Analysis of genes in resident Mφs that are predicted to be activated in the cervix from rats both prepartum on D21 postbreeding and nonpregnant (p<0.01; n = 3/group). No functional annotations met criteria for significant inhibition. See legends to Figs. 3 and 4 for further details.
(TIF)

S2 Fig Ingenuity Pathway Analysis of the proposed network of pathways that reflect expression of Mφ genes in common to the cervix from both prepartum D21 and nonpregnant rats. See Methods and legend to Fig. 5 for graph design details.
(TIF)

S1 Table Increased expression of Mφ genes in cervix from both prepartum and nonpregnant rats. (PDF)

S2 Table Increased expression of Mφ genes in the nonpregnant rat cervix. (PDF)

S3 Table Decreased expression of Mφ genes in the nonpregnant rat cervix. (PDF)
