# Supplementary figures and images for: Macrophage Gene Expression Associated with Remodeling of the Prepartum Rat Cervix: Microarray and Pathway Analyses
Source: PLoS One. 2015 Mar 26;10(3):e0119782. doi: 10.1371/journal.pone.0119782 (PMC4374766; doi:10.1371/journal.pone.0119782)

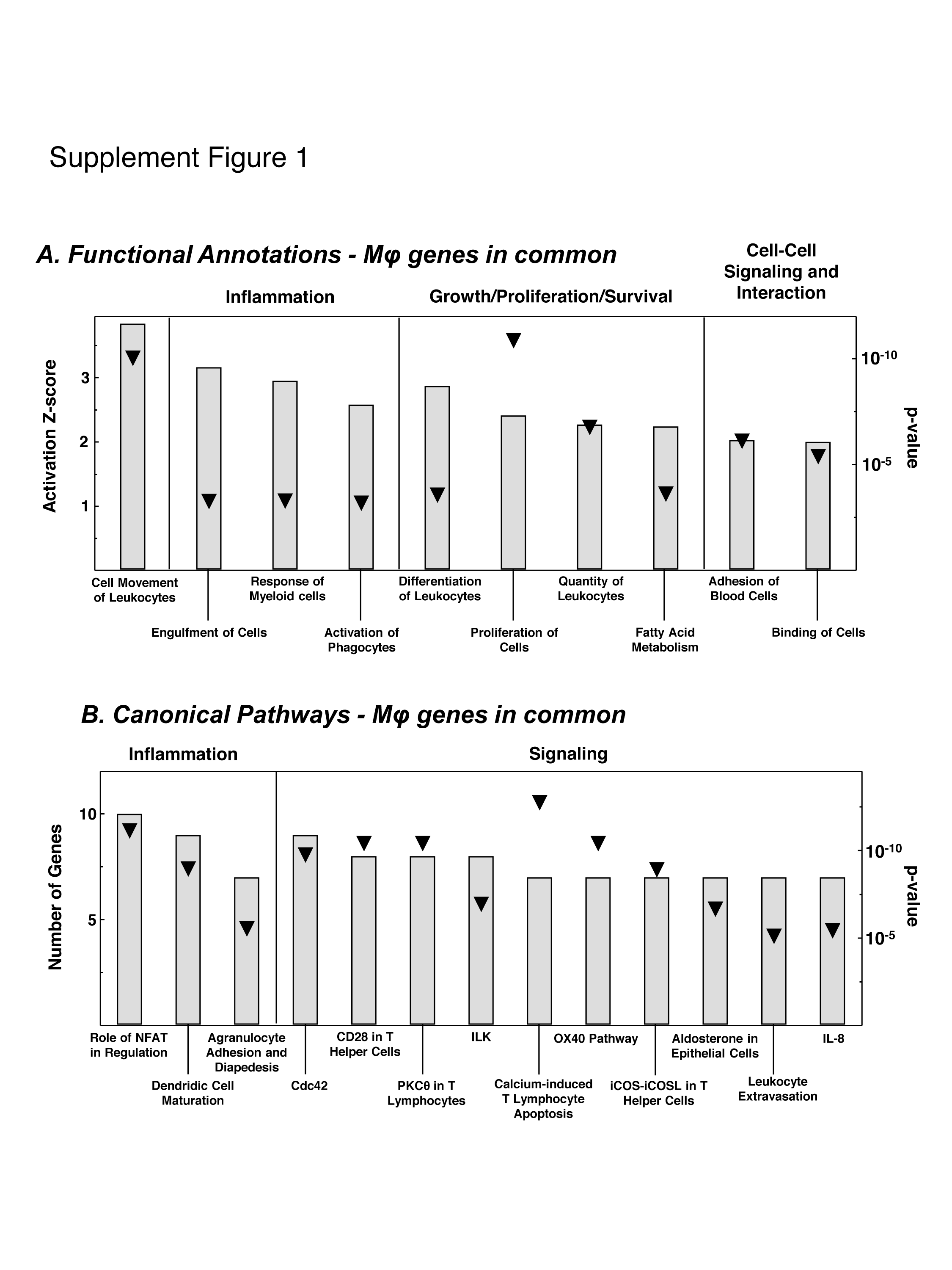

Supplement: S1 Fig — A. Functional annotation categories B. Canonical Pathways from Ingenuity Pathway Analysis of genes in resident Mφs that are predicted to be activated in the cervix from rats both prepartum on D21 postbreeding and nonpregnant (p<0.01; n = 3/group). No functional annotations met criteria for significant inhibition. See legends to Figs. 3 and 4 for further details. (TIF) [file pone.0119782.s001.tif]

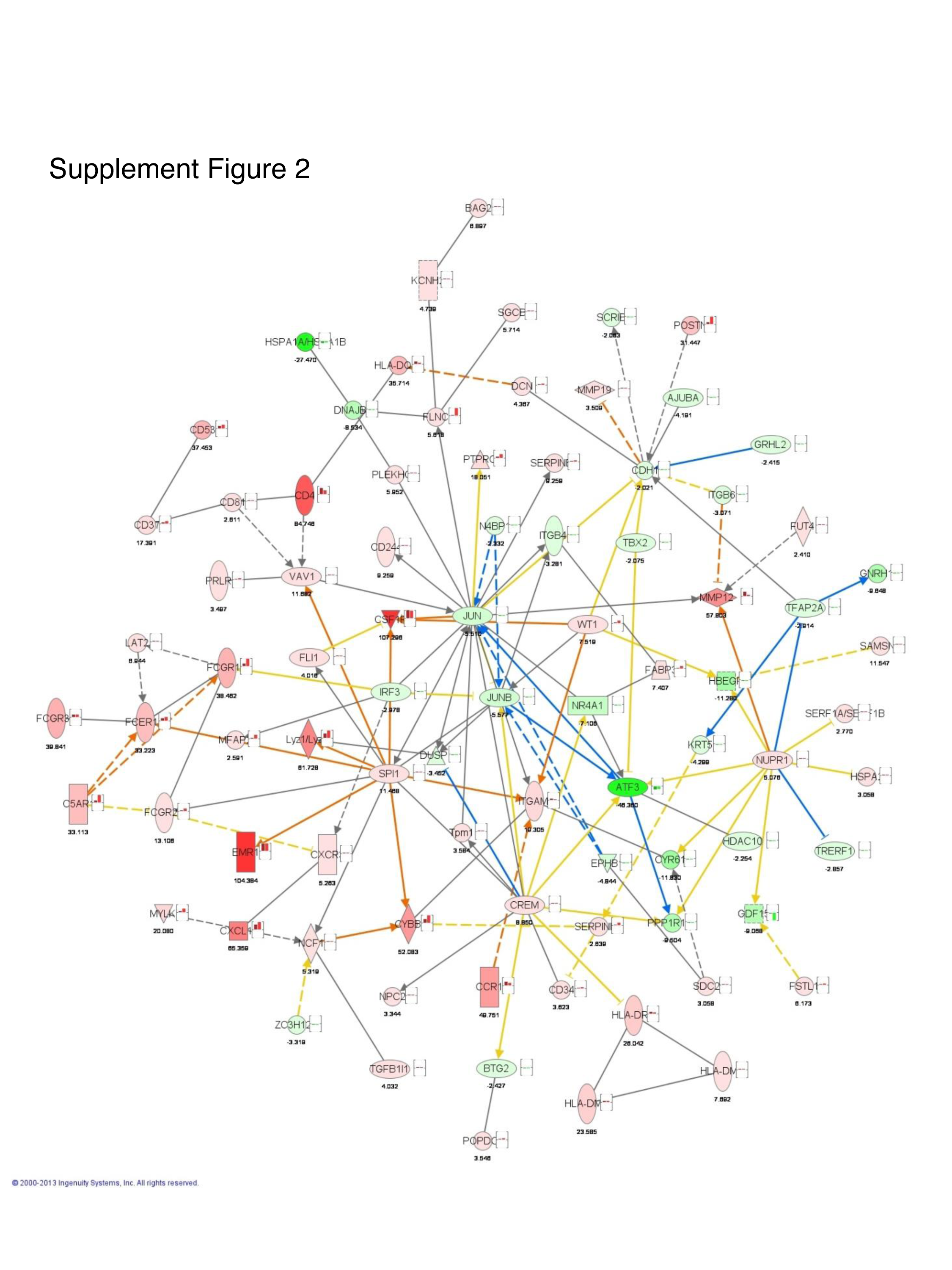

Supplement: S2 Fig — See Methods and legend to Fig. 5 for graph design details. (TIF) [file pone.0119782.s002.tif]
