# Supplementary material for: Macrophage Gene Expression Associated with Remodeling of the Prepartum Rat Cervix: Microarray and Pathway Analyses
Source: PLoS One. 2015 Mar 26;10(3):e0119782. doi: 10.1371/journal.pone.0119782 (PMC4374766; doi:10.1371/journal.pone.0119782)
Supplement: S1 Table — (PDF) [file pone.0119782.s003.pdf]

Supplement Table 1. Increased expression of Mφ genes in cervix from both prepartum (D21) and nonpregnant (NP) rats ($p<0.01$; average whole/Mφ-depleted cervix/group)

Symbol	Entrez Gene Name	Fold Change		Ratio D21/NP
		D21	NP	
CSF1R	colony stimulating factor 1 receptor	107	100	1.073
EMR1	egf-like module containing, mucin-like, hormone receptor-like 1	104	100	1.044
Fcrls	Fc receptor-like S, scavenger receptor	87	100	0.870
CD4	CD4 molecule	85	50	1.695
PLD4	phospholipase D family, member 4	72	100	0.719
CXCL13	chemokine (C-X-C motif) ligand 13	65	100	0.654
Lyz1/Lyz2	lysozyme 2	62	100	0.617
MMP12	matrix metallopeptidase 12 (macrophage elastase)	58	20	2.890
Fcna	ficolin A	56	17	3.390
CYBB	cytochrome b-245, beta polypeptide	52	100	0.521
CCR1	chemokine (C-C motif) receptor 1	50	33	1.493
LY86	lymphocyte antigen 86	49	100	0.488
RGD1562525	similar to cell surface receptor FDFACT	49	100	0.488
FCGR3A	Fc fragment of IgG, low affinity IIIa, receptor (CD16a)	40	33	1.195
FCGR1A	Fc fragment of IgG, high affinity Ia, receptor (CD64)	38	100	0.385
CD53	CD53 molecule	37	50	0.749
HLA-DQB1	major histocompatibility complex, class II, DQ beta 1	36	20	1.786
MS4A6A	membrane-spanning 4-domains, subfamily A, member 6A	35	50	0.702
MS4A7	membrane-spanning 4-domains, subfamily A, member 7	35	50	0.699
FCER1G	Fc fragment of IgE, high affinity I, receptor for; gamma polypeptide	33	50	0.664
C5AR1	complement component 5a receptor 1	33	100	0.331
KCNMB1	K^{+} large conductance Ca^{++}-channel, subfamily M, beta member 1	32	50	0.649
POSTN	periostin, osteoblast specific factor	31	100	0.314
Ms4a6b	membrane-spanning 4-domains, subfamily A, member 6B	31	50	0.617
Ms4a6c	membrane-spanning 4-domains, subfamily A, member 6C	28	50	0.567
LYVE1	lymphatic vessel endothelial hyaluronan receptor 1	28	14	1.928
HSPB7	heat shock 27kDa protein family, member 7 (cardiovascular)	27	100	0.268
HLA-DRA	major histocompatibility complex, class II, DR alpha	26	25	1.042
CLEC4A	C-type lectin domain family 4, member A	26	25	1.036
HLA-DMB	major histocompatibility complex, class II, DM beta	24	20	1.179
ALOX5AP	arachidonate 5-lipoxygenase-activating protein	22	50	0.442
IGSF6	immunoglobulin superfamily, member 6	22	20	1.092
MYLK	myosin light chain kinase	20	50	0.402
GPR183	G protein-coupled receptor 183	20	25	0.802
