# Supplementary material for: Macrophage Gene Expression Associated with Remodeling of the Prepartum Rat Cervix: Microarray and Pathway Analyses
Source: PLoS One. 2015 Mar 26;10(3):e0119782. doi: 10.1371/journal.pone.0119782 (PMC4374766; doi:10.1371/journal.pone.0119782)
Supplement: S2 Table — (PDF) [file pone.0119782.s004.pdf]

Supplement Table 2. Increased expression of Mφ genes in the nonpregnant (NP) rat cervix (p<0.01; average whole/Mφ-depleted cervix/group)

Symbol	Entrez Gene Name	Fold Change
C1QA	complement component 1, q subcomponent, A chain	100
C1QC	complement component 1, q subcomponent, C chain	100
CA3	carbonic anhydrase III, muscle specific	100
COL12A1	collagen, type XII, alpha 1	100
COLEC12	collectin sub-family member 12	100
COX6A2	cytochrome c oxidase subunit VIa polypeptide 2	100
IL13RA2	interleukin 13 receptor, alpha 2	100
TEKT3	tektin 3	100
TNNC2	troponin C type 2 (fast)	100
A2M	alpha-2-macroglobulin	50
ACTG2	actin, gamma 2, smooth muscle, enteric	50
AP1S2	adaptor-related protein complex 1, sigma 2 subunit	50
CFD	complement factor D (adipsin)	50
DES	desmin	50
FIBIN	fin bud initiation factor homolog (zebrafish)	50
FRZB	frizzled-related protein	50
HOXA11	homeobox A11	50
OMD	osteomodulin	50
QPCT	glutaminyl-peptide cyclotransferase	50
SLAMF9	SLAM family member 9	50
SMOC2	SPARC related modular calcium binding 2	50
TMEM100	transmembrane protein 100	50
TWIST2	twist homolog 2 (Drosophila)	50
TYROBP	TYRO protein tyrosine kinase binding protein	50
VCAN	versican	50
ADH1C	alcohol dehydrogenase 1C (class I), gamma polypeptide	33
AIF1	allograft inflammatory factor 1	33
Akr1c14	aldo-keto reductase family 1, member C14	33
C1QB	complement component 1, q subcomponent, B chain	33
CIDEC	cell death-inducing DFFA-like effector c	33
CLEC10A	C-type lectin domain family 10, member A	33
CLEC11A	C-type lectin domain family 11, member A	33
CTSK	cathepsin K	33
DDIT4L	DNA-damage-inducible transcript 4-like	33
GDF10	growth differentiation factor 10	33
HCK	hemopoietic cell kinase	33
HOXA13	homeobox A13	33
HSD11B1	hydroxysteroid (11-beta) dehydrogenase 1	33
INMT	indolethylamine N-methyltransferase	33
KCNIP4	Kv channel interacting protein 4	33
LOX	lysyl oxidase	33
MRGPRF	MAS-related GPR, member F	33
OSR2	odd-skipped related 2 (Drosophila)	33
PF4	platelet factor 4	33
PTGIS	prostaglandin I2 (prostacyclin) synthase	33
RAMP1	receptor (G protein-coupled) activity modifying protein 1	33
RASD2	RASD family, member 2	33
RBP4	retinol binding protein 4, plasma	33
RPRM	reprimo, TP53 dependent G2 arrest mediator candidate	33
RXFP1	relaxin/insulin-like family peptide receptor 1	33
TNFAIP8L2	tumor necrosis factor, alpha-induced protein 8-like 2	33
TNFRSF11B	tumor necrosis factor receptor superfamily, member 11b	33
TREM2	triggering receptor expressed on myeloid cells 2	33
UCMA	upper zone of growth plate and cartilage matrix associated	33
ZP2	zona pellucida glycoprotein 2 (sperm receptor)	33
