# Supplementary material for: Macrophage Gene Expression Associated with Remodeling of the Prepartum Rat Cervix: Microarray and Pathway Analyses
Source: PLoS One. 2015 Mar 26;10(3):e0119782. doi: 10.1371/journal.pone.0119782 (PMC4374766; doi:10.1371/journal.pone.0119782)
Supplement: S3 Table — (PDF) [file pone.0119782.s005.pdf]

Supplement Table 3. Decreased expression of Mφ genes in the nonpregnant (NP) rat cervix ($p<0.01$; average whole/Mφ-depleted cervix/group)

Symbol	Entrez Gene Name	Fold Change
THSD4	thrombospondin, type I, domain containing 4	-37
FOS	FBJ murine osteosarcoma viral oncogene homolog	-21
HR	hairless homolog (mouse)	-11
TP73	tumor protein p73	-10
SELE	selectin E	-8
CDCA7L	cell division cycle associated 7-like	-7
PAX2	paired box 2	-7
PLEK2	pleckstrin 2	-7
ARHGAP32	Rho GTPase activating protein 32	-7
Gm884	predicted gene 884	-6
GADD45B	growth arrest and DNA-damage-inducible, beta	-6
TIAM1	T-cell lymphoma invasion and metastasis 1	-6
PTPRF	protein tyrosine phosphatase, receptor type, F	-6
SEMA3F	sema domain,immunoglobulin domain(Ig),short basic domain,secreted,(semaphorin) 3F	-5
IFFO2	intermediate filament family orphan 2	-5
DLL1	delta-like 1 (Drosophila)	-5
Terc	telomerase RNA component	-5
SYTL1	synaptotagmin-like 1	-5
MARK2	MAP/microtubule affinity-regulating kinase 2	-5
CAMSAP3	calmodulin regulated spectrin-associated protein family, member 3	-5
SELP	selectin P (granule membrane protein 140kDa, antigen CD62)	-5
B4GALNT1	beta-1,4-N-acetyl-galactosaminyl transferase 1	-5
CKMT1A/B	creatine kinase, mitochondrial 1B	-5
JAG1	jagged 1	-5
EPHB3	EPH receptor B3	-5
GNAT1	guanine nucleotide binding protein (G protein), alpha transducing activity polypeptide 1	-5
ULK1	unc-51-like kinase 1 (C. elegans)	-5
ZNF692	zinc finger protein 692	-5
RGS12	regulator of G-protein signaling 12	-5
KCNJ14	potassium inwardly-rectifying channel, subfamily J, member 14	-5
